# MicroRNA-488 inhibits proliferation and motility of tumor cells via downregulating FSCN1, modulated by Notch3 in breast carcinomas

**DOI:** 10.1038/s41419-020-03121-5

**Published:** 2020-10-24

**Authors:** Yang Wu, Ming-Heng Yuan, Hua-Tao Wu, Wen-Jia Chen, Man-Li Zhang, Qian-Qian Ye, Jing Liu, Guo-Jun Zhang

**Affiliations:** 1 Changjiang Scholar’s Laboratory/Guangdong Provincial Key Laboratory for Diagnosis and Treatment of Breast Cancer, Shantou University Medical College, 515041 Shantou, China; 2 Department of General Surgery, the First Affiliated Hospital of Shantou University Medical College, 515041 Shantou, China; 3 Department of Physiology/Cancer Research Center, Shantou University Medical College, 515041 Shantou, China; 4 Department of Breast and Thyroid, Xiang’an Hospital of Xiamen University, 361101 Xiamen, China

**Keywords:** Breast cancer, Breast cancer

## Abstract

As important modulators in multiple physiological processes, microRNAs (miRNAs) have been reported in various malignant tumors, including breast cancer. The current study investigated the function of a new tumor suppressor microRNA, miR-488, and its molecular mechanism of metastasis in breast cancers. CCK8 and transwell assays revealed that the upregulated miR-488 level significantly inhibited the proliferation and migration of breast cancer cells. As a potential downstream gene, the mRNA and protein level of FSCN1 was suppressed by increased miR-488 and vice versa. Luciferase assay showed that miR-488 directly bind to the 3′UTR of FSCN1 and suppressed the translation process of FSCN1. The promoter region of miR-488 was directly bound by Notch3 and promoted the expression of miR-488 transcriptionally. Immunohistochemistry results revealed that in patients with breast cancer, the expression of Notch3 and were negatively correlated with the FSCN1 levels significantly. Therefore, the current finding predicted miR-488 as a tumor suppressor molecule in breast cancer, and demonstrated that Notch3/miR-488/FSCN1 axis is established and involved in regulating the metastasis of breast cancers, providing novel therapeutic targets for patients with breast cancers.

## Introduction

Breast cancer is the leading cause of cancer death in women younger than 45 years old, while metastasis of tumor cells is the main cancer-related death in patients^1^. The early-stage patients with standardized treatments could have a 5-year survival rate as high as 98%, whereas those late-stage patients with metastasis only have a 26% survival rate^2^. Although oncoplastic surgery provided a new choice for young breast cancer patients who have to face their social and personal pressure, the metastasis is still one of the undeniable contraindications of oncoplastic surgery^3^. The mechanisms of breast cancer metastasis are still unclear and need to be investigated further.

On the other hand, microRNAs (miRNAs), a set of small noncoding RNAs, was reported to exert diverse and important effects on the development and metastasis of cancers, of which miR-488 was first reported to suppress the proliferation of prostatic cancer cell via downregulating mRNA and protein levels of androgen receptor^4^. In non-small cell lung cancer, miR-488 activated eIF3a-mediated NER signal pathway, sequentially inhibited the proliferation and cisplatin resistance of tumor cells^5^. The proliferation and invasion abilities of hepatocellular carcinoma cells were also inhibited by the high-level of miR-488 through the downregulation of ADAM9 and lncRNA HULC^6^. In tongue squamous carcinoma, miR-488 could increase the sensitivity of tumor cells to chemotherapy^7^, supposed that miR-488 was involved in the inhibition of the epithelial–mesenchymal transition (EMT) process^8^. Recently, Takaaki et al. demonstrated that circulating pre-miRNA-488 in peripheral blood would be a potential biomarker for predicting recurrence of breast cancer^9^. Although, these findings suggested miR-488 as tumor suppressor factor in various cancers, the function of miR-488 in breast cancer and its underlying mechanism was unclear, prompting further investigation of the correlation between miR-488 and breast cancer.

In this study, miR-488 is the main study focus and revealed its related signal pathway involved in the process of breast cancer, providing a potential novel therapeutic target for patients with breast cancers.

## Materials and methods

### Cell culture, oligonucleotides, plasmids, and transfection

Human breast cancer cell lines, MCF-7, T47D, SKBR3, MDA-MB-231, and BT-549 were purchased from American Type Tissue Collection (ATCC) and cultured in DMEM medium supplemented with 10% fetal bovine serum (FBS) and 1% penicillin/streptomycin. Normal human breast cancer cells, hMEC and MCF-10A were also purchased from ATCC, and cultured in MEPiCM medium (Cat7611, ScienCell) supplemented with MEPiCGs (Cat7652, ScienCell), 10% FBS, and 1% penicillin/streptomycin.

The oligonucleotides of the miR-488 mimics/inhibitors, siNotch3, siFSCN1, and their corresponding negative control (NC; Supplementary Table 1) were purchased from GenePharma (Shanghai, China).

To overexpress the intracellular domain of Notch3 (N3ICD), pCLE-N3ICD (Plasmid #26894), and vector pCLE (Plasmid #17703) were obtained from Addgene (Cambridge, MA, USA)^10^. The plasmids of pEGFP-FSCN1 and vector pEGFP-N1, as well as pGLC-FSCN1-3′UTR (3′-untranslated region) and vector pGLC (short name for pGL3-Control purchased from Promega), were gifts from Professor Liyan Xu^11^. The reporter gene with the core region of the miR-488 promoter, pGLE-miR-488-pro were constructed into the HindIII/XhoI sites of the luciferase reporter vector pGLE (short name for pGL3-Enhancer purchased from Promega). The mutant type of miR-488 promoter with the deletion of the CSL-binding site was created in the same vector, named as pGLE-miR-488-pro-Mut.

The miR-488 mimics/inhibitors, DNA plasmids, and/or siRNA were transfected in breast cancer cells with lipofectamine 3000 (Thermo Fisher, MA, USA). Cells were collected 48 h after transfection for further experiments.

### RNA purification, reverse transcription, and real-time PCR performance

Total RNAs, including mRNAs and miRNAs, were extracted from cancer cells using Trizol Total RNA Isolation Reagent (Invitrogen, CA, USA) according to the manufacturer’s instructions. Reverse transcription was conducted using Prime ScriptTM RT Reagent Kit DRR036A and DRR047A (TAKARA, Japan) to synthesize cDNAs for mRNAs and miRNAs, respectively. Real-time PCR was performed with SYBR Select Master Mix (Thermo Fisher, MA, USA) on the CFX96 Real-time PCR Detection System (Bio-Rad, CA, USA) to detect the expression levels of mRNAs and miRNAs. The sequences of primers used were listed in Supplementary Table 2.

### Protein extraction and western blot

The whole protein of cancer cells was extracted with RIPA lysis buffer (Millipore, USA), analyzed using western blots and visualized on ChemiDoc XRS + (Bio-Rad, USA), as described previously^12^. Primary antibodies used and volume dilution was listed in Supplementary Table 3.

### Cell counting kit-8 assays

Forty eight hours after transfection, the transfected MDA-MB-231 (1 × 10^3^/well) or MCF-7 (3 × 10^3^/well) cells were plated into 96-well plates, with 200 μL 10% FBS/DMEM. Cell counting kit-8 (CCK8) reagent was added to the medium 0, 24, 48, 72, 96, and 120 h after plating. Two hours after that, absorbance was measured at 490 nm using a microplate reader, SpectraMax M5 (Sunnyvale, CA, USA).

### Colony formation assays

The transfected MDA-MB-231 (1,000 cells/well) or MCF-7 (500 cells/well) cells were plated in six-well plates with 2 mL 10% FBS/DMEM. Two to three weeks later, the number of cell clones was calculated under a Zeiss microscope (Zeiss, Oberkochen, Germany) with crystal violet staining.

### Wound healing assays

Forty eight hours after transfection, cells cultivated in 12-well plates at 90% confluence were scratched a 2-mm-wide wound by 200 μL tips and cultured in FBS-free DMEM. The width of the wound was taken as a picture at 0 and 24 (MDA-MB-231) or 48 h (MCF-7) after scratching. The healing abilities of tumor cells were measured and evaluated by the widths of injury lines under the microscope a Zeiss microscope (Zeiss, Oberkochen, Germany; magnificantion: 40×).

### Transwell assays

To assess the cellular migration and invasion abilities, transwell assays were performed using a 24-well chamber, partitioned by a polycarbonate membrane (8-μm pore size) without or with Matrigel (BD, Franklin Lakes, NJ, USA). Forty eight hours after transfection, breast cancer cell lines, MDA-MB-231 (2.0 × 10^4^ cells/well) or MCF-7 (5.0 × 10^4^ cells/well) were seeded into the upper chamber with FBS-free DMEM, while 10% FBS/DMEM was added into the lower chamber. The cells were allowed to migrate for 24 (MDA-MB-231) or 36 h (MCF-7), and invade for 36 (MDA-MB-231) or 48 h (MCF-7) at 37 °C in a humidified atmosphere containing 5% CO_2_. After removing the cells on the upper side of the membrane, the cells on the lower side of the membrane were stained with 0.1% crystal violet and counted by two individual investigators under the microscope a Zeiss microscope (Zeiss, Oberkochen, Germany; magnificantion: 200×).

### Bioinformatics analyses

The potential targets of miR-488 was predicted by miRDB (http://mirdb.org/)^13^, TargetScan 7.2 (http://www.targetscan.org/vert_72/)^14^, and miRanda (http://www.miranda.org/). The overlaps of potential targets of miR-488 were conducted by Venn diagrams (http://bioinformatics.psb.ugent.be/webtools/Venn/). The promoter region of miR-488 was searched and downloaded from UCSC (http://genome.ucsc.edu/)^15^. The potential transcription factor to regulate miR-488 was analyzed using AnimalTFDB (http://bioinfo.life.hust.edu.cn/AnimalTFDB/#!/)^16^.

### Dual-luciferase reporter assays

To investigate the regulating function of miR-488 on FSCN1, cells were co-transfected with pGLC-FSCN1-3′UTR or pGLC-FSCN1-3′UTR-Mut, miR-488 mimic or inhibitor, and pRL-SV40 (Promega, USA), using Lipofectamine 3000 (Invitrogen, USA). And to explore the transcriptional regulation of miR-488 expression, cells were co-transfected with pGLE-miR-488-pro or pGLE-miR-488-pro-Mut, pCLE-N3ICD or pCLE (vector), and pRL-SV40 (Promega, USA), using Lipofectamine 3000 (Invitrogen, USA). Luciferase and Renilla signals were examined 36 h after transfection using a Dual-Luciferase Reporter Assay Kit (E1910, Promega) according to the manufacturer’s protocol.

### Chromatin immunoprecipitation assay

Chromatin immunoprecipitation (ChIP) assay was performed according to the laboratory manual^17^. Breast cancer cells were fixed with 1% formaldehyde to covalently crosslink protein to DNA. Crosslinked DNA was harvested, sheared with sonication, and subjected to immunoprecipitation for overnight at 48 °C with anti-Notch3 antibody or IgG. Finally, PCR was conducted to measure the enrichment of DNA fragments in the putative Notch3/CSL-binding sites in the promoter region of miR-488. Sequences of primers and all the antibodies used in ChIP assays are showed in Supplementary Tables 2 and 3, respectively.

### Patient information and ethics statement

To evaluate the expression pattern of Notch3/miR-488/FSCN1 axis in clinic, 106 patients with breast cancer had undergone surgery at the Cancer Hospital of Shantou University Medical College between Feb 2013 and Jan 2014, without evidence of metastasis at the first visit. The informed consent was obtained from all patients. The use of human tissues in this study was approved by the Academic Committee of Shantou University Medical College. This study was conducted according to the principles expressed in the Declaration of Helsinkin.

### Immunohistochemistry

Surgical specimens of breast cancer tissues were formalin-fixed, paraffin-embedded, and cut into 4-μm-thick sections. Deparaffinization, rehydration, epitope retrieval, and inactivation of endogenous peroxidase activity was achieved as described^18^. Samples were incubated overnight at 4 °C with anti-Notch3 or anti-FSCN1, then visualized using 3,3′-diaminobenzidine tetrahydrochloride. Sections without primary antibody were used as NCs. Counterstaining was performed by hematoxylin.

Results were evaluated independently by two investigators under a bright-field microscope (Olympus, Japan; magnification: 200× and 400×) with no prior knowledge of the patients data. Pathological scoring were performed as previsouly described^17^.

### Statistical analysis

Each experiment was repeated at least three times. The statistical analyses were performed using Student’s *t* test and one-way ANOVA. Levels of statistical significance were evaluated with data using chi-square test or Fisher’s exact test for categorical variables. The correlation of Notch3 and FSCN1 expression was conducted by Spearman rank correlation test. Statistical significance was assessed and considered at *p* value < 0.05.

## Results

### The expression of miR-488 was decreased in breast cancer cell lines, compared with normal breast cells, especially in triple-negative breast cancer cells

**Fig. 1:**
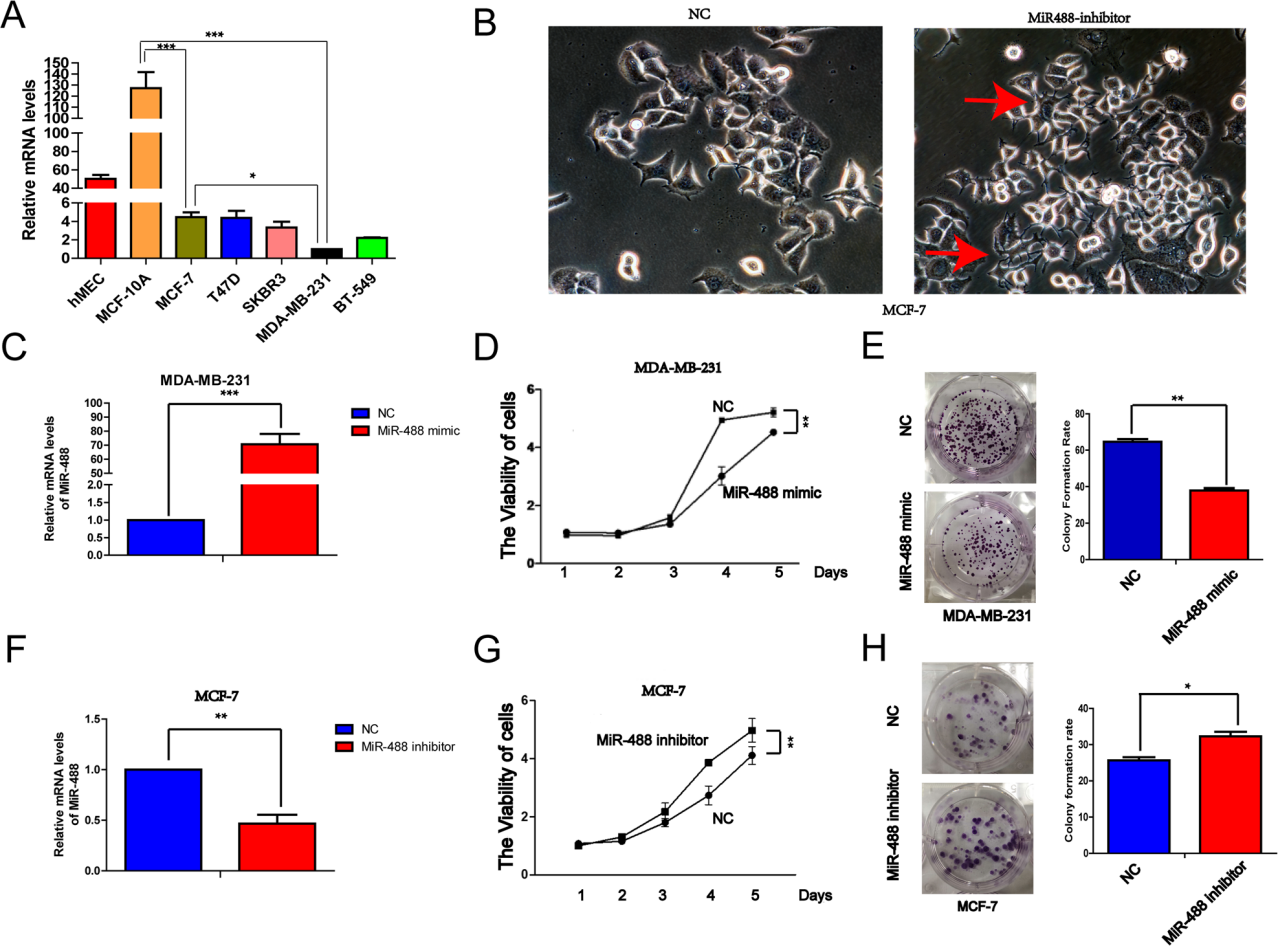
The expression pattern of miR-488 in breast cancer cell lines and its effect on the proliferation rate of breast cancer cells. **A** The expression of miR-488 was significantly decreased in all examined breast cancer cell lines, especially in triple-negative breast cancer (TNBC) cells. **B** The suppression of endogenous miR-488 in MCF-7 cells significantly changed their morphology with obvious pseudopodia stretching out (red arrows). **C** The increased expression of miR-488 was conducted through transfecting miR-488 mimic. **D** The proliferation rate of MDA-MB-231 cells was significantly suppressed with exogenous miR-488 by a miR-488 mimic. **E** The number of cell colonies was decreased in MDA-MB-231 cells treated by a miR-488 mimic. **F** The decreased expression of miR-488 was performed through transfecting miR-488 inhibitors. **G** The proliferation rate of MCF-7 cells was promoted by miR-488 inhibitors to suppressing the endogenous miR-488. **H** The number of MCF-7 cell colonies was formed more in the miR-488 inhibitor-treated group than that in negative control (NC) cells. *p < 0.05, **p < 0.01, and ***p < 0.001.

To explore the expression pattern of miR-488 in breast cell lines, total RNA in two normal breast cells (hMEC and MCF-10A) and five breast cancer cell lines (MCF-7, T47D, SKBR3, MDA-MB-231, and BT-549) were collected. Real-time PCR results revealed that the level of miR-488 was significantly decreased in breast cancer cell lines compared with normal breast cells. Interestingly, the expression of miR-488 in aggressive triple-negative breast cancer (TNBC) cells (MDA-MB-231 and BT-549) was even less than that in luminal breast cancer cells (MCF-7 and T47D; Fig. 1A), predicting that miR-488 may be a favorable factor in preventing metastasis process.

### MiR-488 inhibited the proliferation of breast cancer cells

To investigate the function of miR-488 on breast cancers, miR-488 mimic and inhibitor were transfected into MDA-MB-231 and MCF-7 cells, respectively (Fig. 1C, F). After suppressing endogenous miR-488 levels in MCF-7 cells using miR-488 inhibitor, the morphology of MCF-7 was changed with obvious pseudopodia stretching out, which may enhance invasiveness and motility of cancer cells (Fig. 1B).

CCK8 assays showed that enforced-overexpressing miR-488 in MDA-MB-231 reduced their proliferation rate appropriate 27%, compared with NC (Fig. 1D), while the proliferation rate of MCF-7 cells with miR-488 inhibitor was significantly increased as 1.6 times as that of MCF-7 cells with NC (Fig. 1G). The subsequent colony-forming assays were also revealed that miR-488 mimic increased the miR-488 level and suppressed the formation of the MDA-MB-231 colony (Fig. 1E) and BT-549 colony (Supplementary Fig. S1A), while inhibition of endogenous miR-488 promoted the colony formation of MCF-7 cells (Fig. 1H) and T47D cells (Supplementary Fig. 1B).

### MiR-488 inhibited the migration and invasion of breast cancer cells

**Fig. 2:**
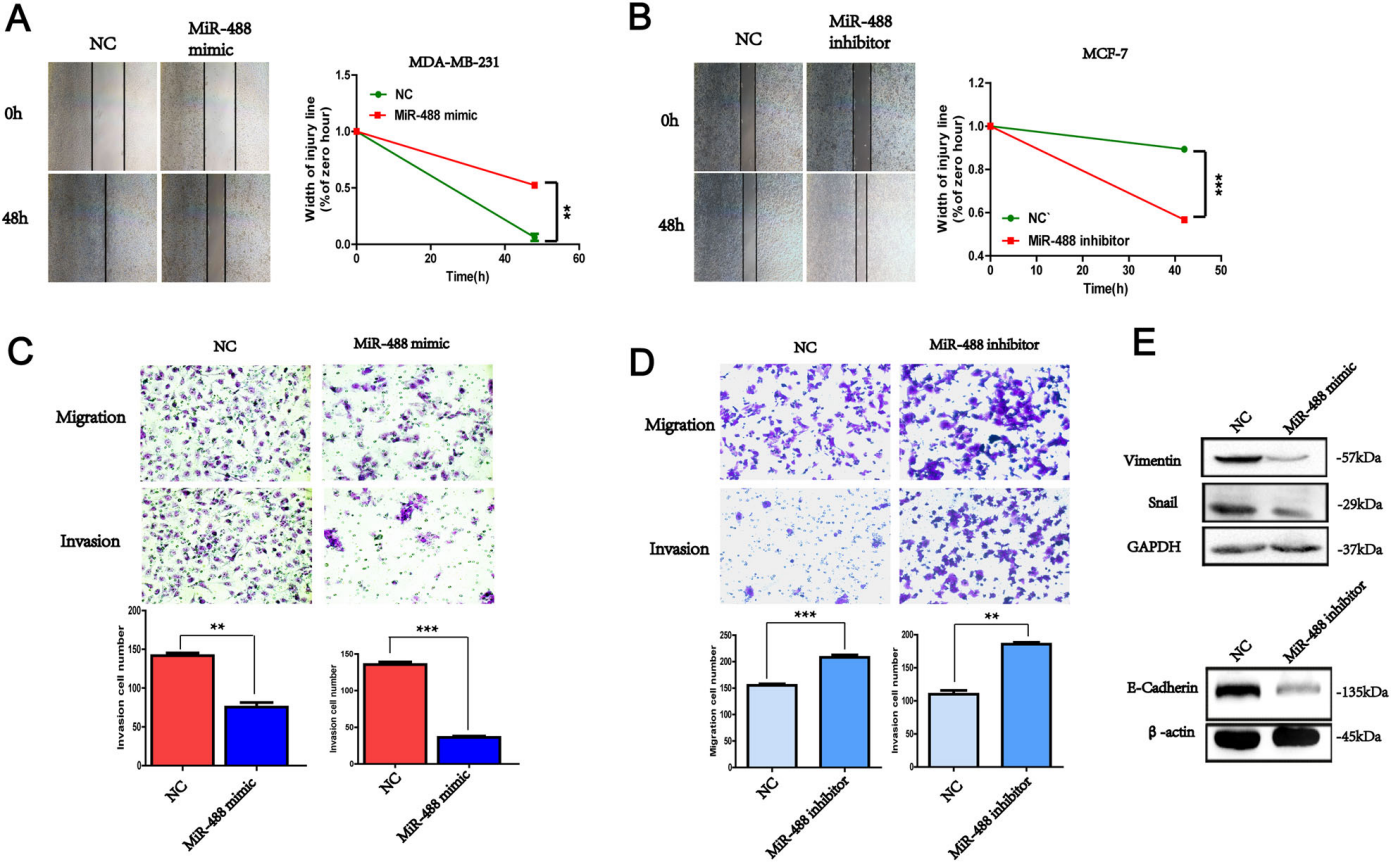
The miR-488 inhibited the motility of breast cancer cells. **A** The wound healing ability in MDA-MB-231 with miR-488 mimic was weak with a large width compared with NC. **B** In MCF-7 cells, the miR-488 inhibitor promoted the wound healing ability with a short width. **C** In transwell assay, miR-488 mimic significantly suppressed the migration and invasion abilities of MDA-MB-231. **D** The cells penetrated through the membrane was increased of MCF-7 cell with miR-488 inhibitor. **E** The mesenchymal marker, Vimentin, and Snail expression were suppressed by increased miR-488 level and the epithelial marker, E-cadherin was increased by miR-488 inhibitor. *p < 0.05, **p < 0.01, and ***p < 0.001.

To investigate the function of miR-488 on motility of tumor cells, wound healing assay was conducted and found that exogenous miR-488 mimic extended the wound healing time of MDA-MB-231 cells compared with control group (Fig. 2A), while miR-488 inhibitor downregulated the endogenous miR-488 levels and promoted the wound healing ability of MCF-7 cells (Fig. 2B). Transwell assays were conducted to investigate the motility of tumor cells and demonstrated that artificially overexpression of miR-488 significantly suppressed the migration (matrigel-uncoated) and invasion (matrigel-coated) ability of MCF-7 cells (Fig. 2C), while miR-488 inhibitor dramatically increased the migration and invasion ability of MDA-MB-231 cells (Fig. 2D). To explore the potential mechanism, the expression of EMT markers was examined and found that the mesenchymal markers, Vimentin, and Snail level were negatively associated with the high miR-488 expression in MDA-MB-231 cells, and the epithelial marker, E-cadherin was suppressed by the miR-488 inhibitor in MCF-7 cells (Fig. 2E).

### The expression of FSCN1 was regulated by miR-488

**Fig. 3:**
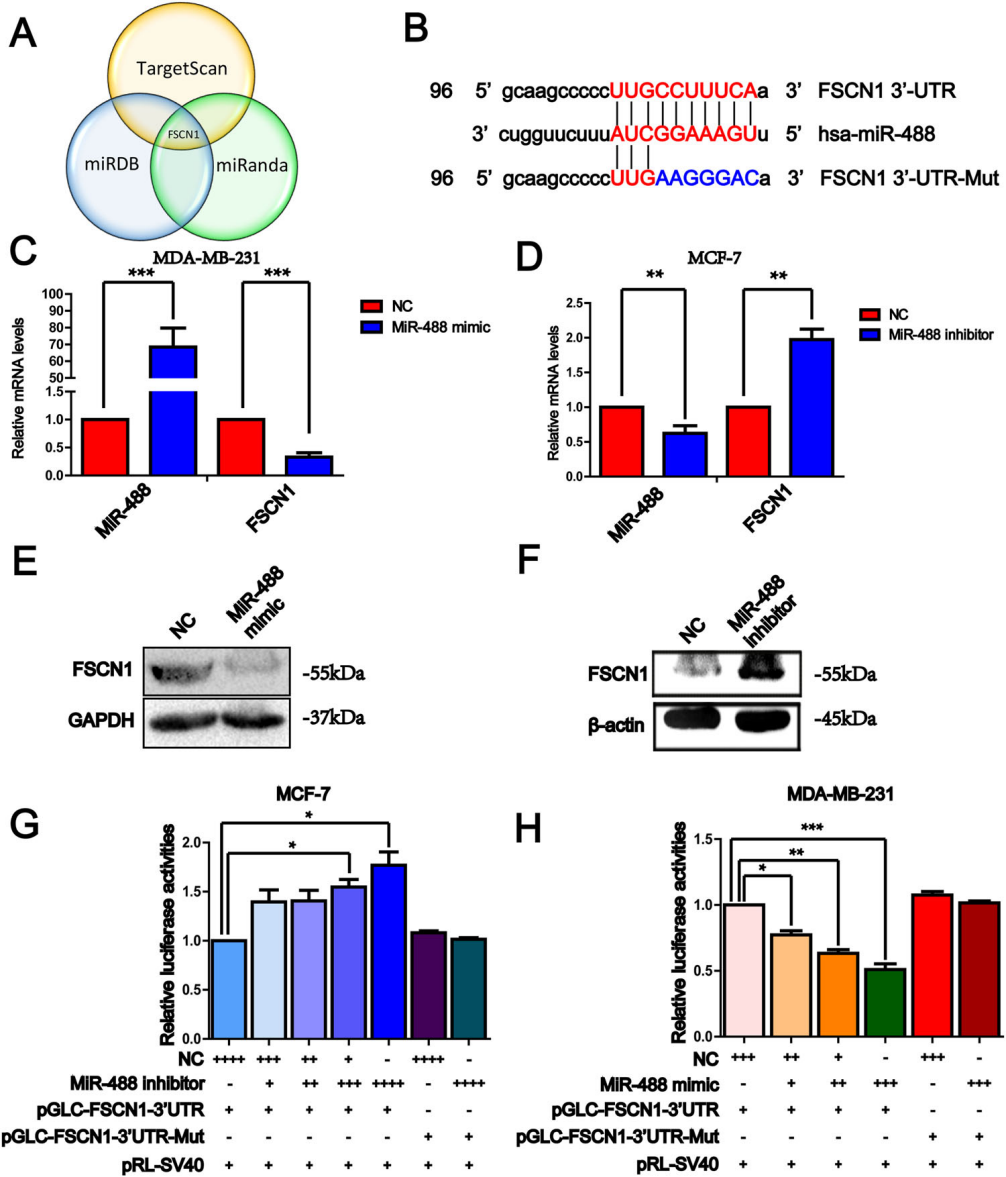
FSCN1 was the potential target of miR-488, and its expression was regulated by miR-488. **A** The potential target genes of miR-488 were predicted by online tools. **B** The schematic showed the seed sequence of miR-488 and its potential binding site in the 3′-untranslated region (3′UTR) of FSCN1. **C**, **E** The mRNA (**C**) and protein (**E**) level of FSCN1 was significantly suppressed by a miR-488 mimic. **D**, **F** The mRNA (**D**) and protein (**F**) level of FSCN1 was significantly upregulated by suppressed miR-488 level. **G** In MCF-7 cell, the miR-488 inhibitor significantly upregulated the luciferase activities of the FSCN1-3′UTR reporter gene in a dose-dependent manner, while that in the mutant group was not changed. **H** In MDA-MB-231 cells, luciferase activities of the FSCN1-3′UTR reporter gene were suppressed by increased miR-488 using mimic in a dose-dependent manner and not changed in the mutant reporter gene. *p < 0.05, **p < 0.01, and ***p < 0.001.

To explore the downstream target of miR-488 in breast cancer, online bioinformatics databases were used to predict the potential target genes and found FSCN1 as a potential target of miR-488 (Fig. 3A). After searching the sequence of FSCN1, its 3′UTR contains a conservatively binding site of miR-488, as a strong potential regulating target (Fig. 3B).

As expected, the mRNA and protein level of FSCN1 was significantly downregulated in MDA-MB-231 cells by miR-488 mimic (Fig. 3C, E). And in MCF-7 cells, the miR-488 inhibitor dramatically increased the expression of FSCN1 in transcriptional and translational levels (Fig. 3D, F).

### MiR-488 directly bind to the core sequence in the 3′UTR of FSCN1 to regulate the expression of FSCN1

As expected, the cytoskeletal protein, FSCN1 was proved to be involved in the mechanism of metastasis of breast cancer via promoting EMT process (Supplementary Fig. 2). To explore the mechanism of miR-488 regulating FSCN1, the reporter genes with the 3′UTR of FSCN1, pGLC-FSCN1-3′UTR, and mutant pGLC-FSCN1-3′UTR-Mut (Fig. 3B) were constructed to. In MCF-7 cells, luciferase assay results showed that the luciferase activities were increased by knockdown miR-488 using its inhibitor in a dose-dependent manner. However, the luciferase activities had no changes in the mutant group with decreased miR-488 levels (Fig. 3G). To confirm the regulation of miR-488 on the 3′UTR region of FSCN1, miR-488 mimic was used in MDA-MB-231 cells, and co-transfected with the reporter gene pGLC-FSCN1-3′UTR. The luciferase activities were significantly decreased by increasing miR-488 levels in a dose-dependent manner and had no change in the mutant group (Fig. 3H).

### The inhibition of proliferation of breast cancer cells by miR-488 can be attenuated by its target, FSCN1

**Fig. 4:**
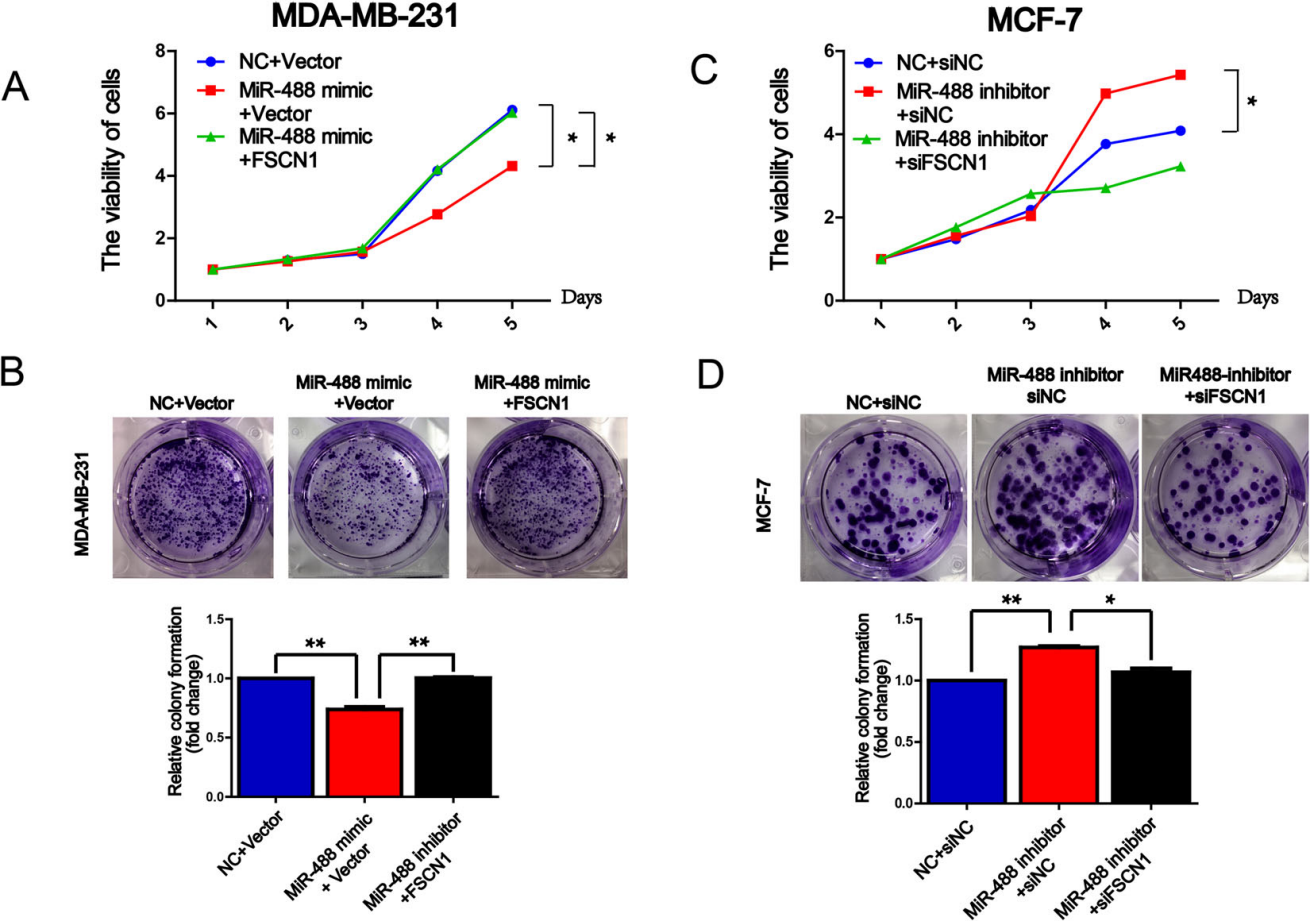
The suppressive function of miR-488 on proliferation was recovered by re-overexpressing FSCN1 artificially. **A** The proliferation rate of MDA-MB-231 was significantly suppressed by miR-488 mimic and the effect was recovered by reexpressing FSCN1 to promote the proliferation rate. **B** The colony formation assay showed that the suppressive effect of miR-488 was reversed by overexpressing FSCN1 in MDA-MB-231-miR-488 mimic cells. **C** Conversely, the miR-488 inhibitor promoted the proliferation rate of MCF-7, which was repressed by knockdown FSCN1 at the same time. **D** The number of MCF-7 cell colonies was increased dramatically with miR-488 inhibitor, and the promoted effect of miR-488 inhibitor was suppressed by siFSCN1. *p < 0.05, **p < 0.01, and ***p < 0.001.

After examination, the FSCN1 expression was increased by suppressing the miR-488 levels, and the increasing effect was suppressed by siFSCN1 in the miR-488 inhibitor-treated MCF-7 cells accordingly (Supplementary Fig. 3). In MDA-MB-231 cells, the inhibition effect on the proliferation of high miR-488 levels was attenuated by overexpression of FSCN1 (Fig. 4A). In Fig. 4B, the colony formation in cells with miR-488 mimic was significantly decreased, and the re-overexpression of FSCN1 in such cells increased the formation of the colony reversely. Conversely, the proliferation rate of MCF-7 cells was promoted by miR-488 inhibitor and recovered by suppression FSCN1 at the same time (Fig. 4C). Colony assays revealed that siFSCN1 suppressed the subsequent-increasing FSCN1 in MCF-7 cells treated by miR-488 inhibitor, and significantly decreased the formation of tumor colonies (Fig. 4D).

### The inhibition of motility of breast cancer cells by miR-488 can be attenuated by its target, FSCN1

**Fig. 5:**
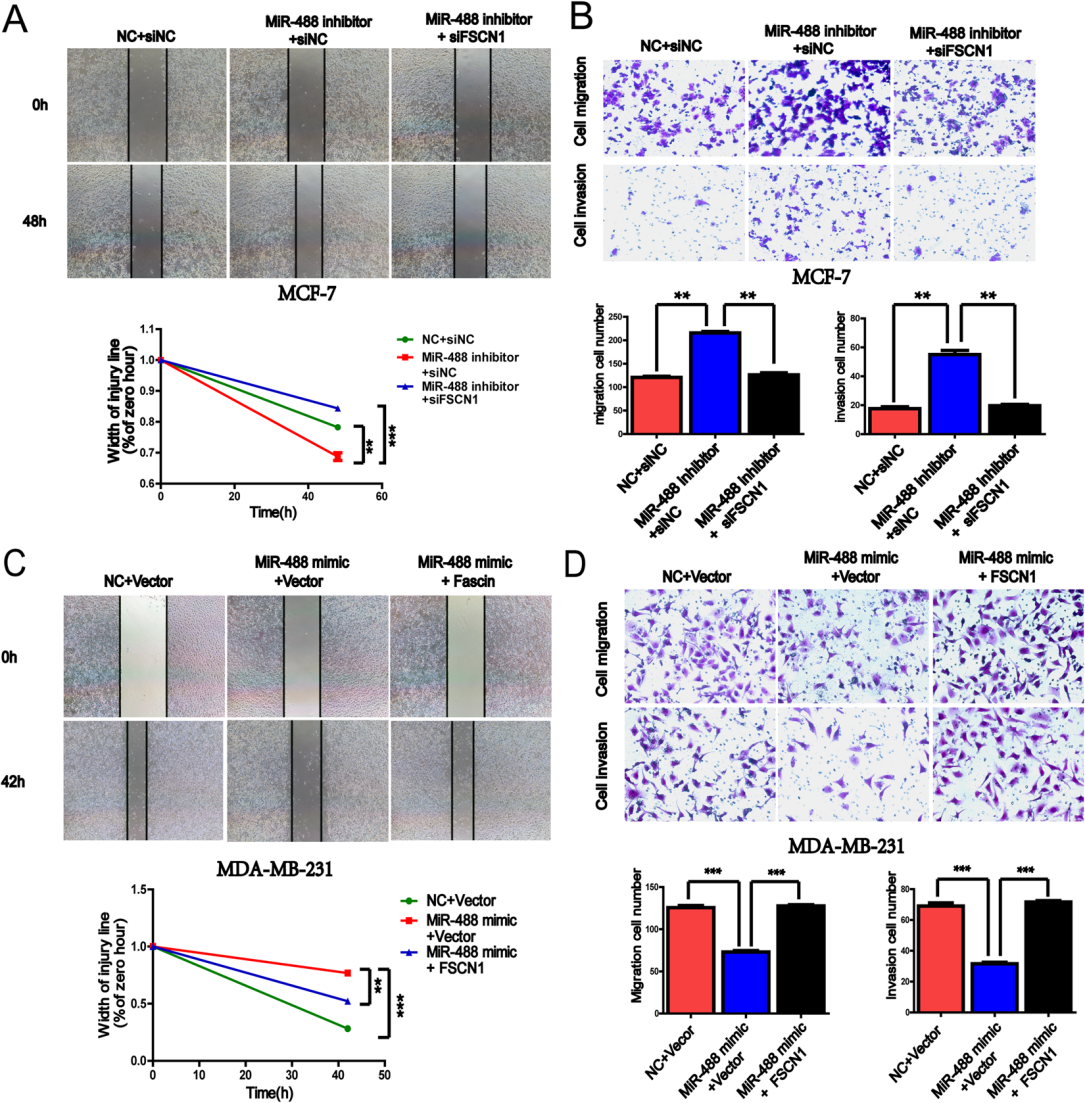
The inhibition of motility of breast cancer cells by miR-488 can be attenuated by its target, FSCN1. **A** The wound width was decreased under the treatment of miR-488 inhibitor, consistent with the level of FSCN1, and the wound healing abilities was resuppressed when knockdown the expression of FSCN1 in MCF-7-miR-488-inhibitor cells. **B** The migration and invasion abilities were consistent with the wound healing abilities, reflecting the motility of MCF-7 cells, which was suppressed in siFSCN1+miR-488 inhibitor-treated cell compared with miR-488 suppressing function. **C** The wound healing rate was decreased by miR-488 mimic, and the reverse effect was found in the re-overexpression of FSCN1 in MDA-MB-231 cells. **D** The migration and invasion abilities were consistent with the wound healing assays, predicting the motility of MDA-MB-231, which was promoted by re-overexpression of FSCN1 compared with the NC group.*p < 0.05, **p < 0.01, and ***p < 0.001.

In wound healing assays, although the wound recovered faster in the miR-488 inhibitor group than that in the NC group, the suppression of FSCN1 inhibited the cell motility and restored the large scratching wound (Fig. 5A). The similar results were also found in transwell assay experiments, that both of the migratory and invasive abilities of breast cancer cells were reversed by downregulating FSCN1 expression in the miR-488 inhibitor group (Fig. 5B).

Conversely, in MDA-MB-231 cells with miR-488 mimic, overexpression of FSCN1 attenuated the miR-488-induced inhibition of wound healing rate (Fig. 5C). The rescue experiments were also conducted in transwell assays and revealed that the breast cancer cells transfected with miR-488 mimic, and pEGFP-FSCN1 showed more aggressive motility than the ones with miR-488 and control vector (Fig. 5D).

### Notch3 directly bind to the promoter region of miR-488 and upregulated its expression level

**Fig. 6:**
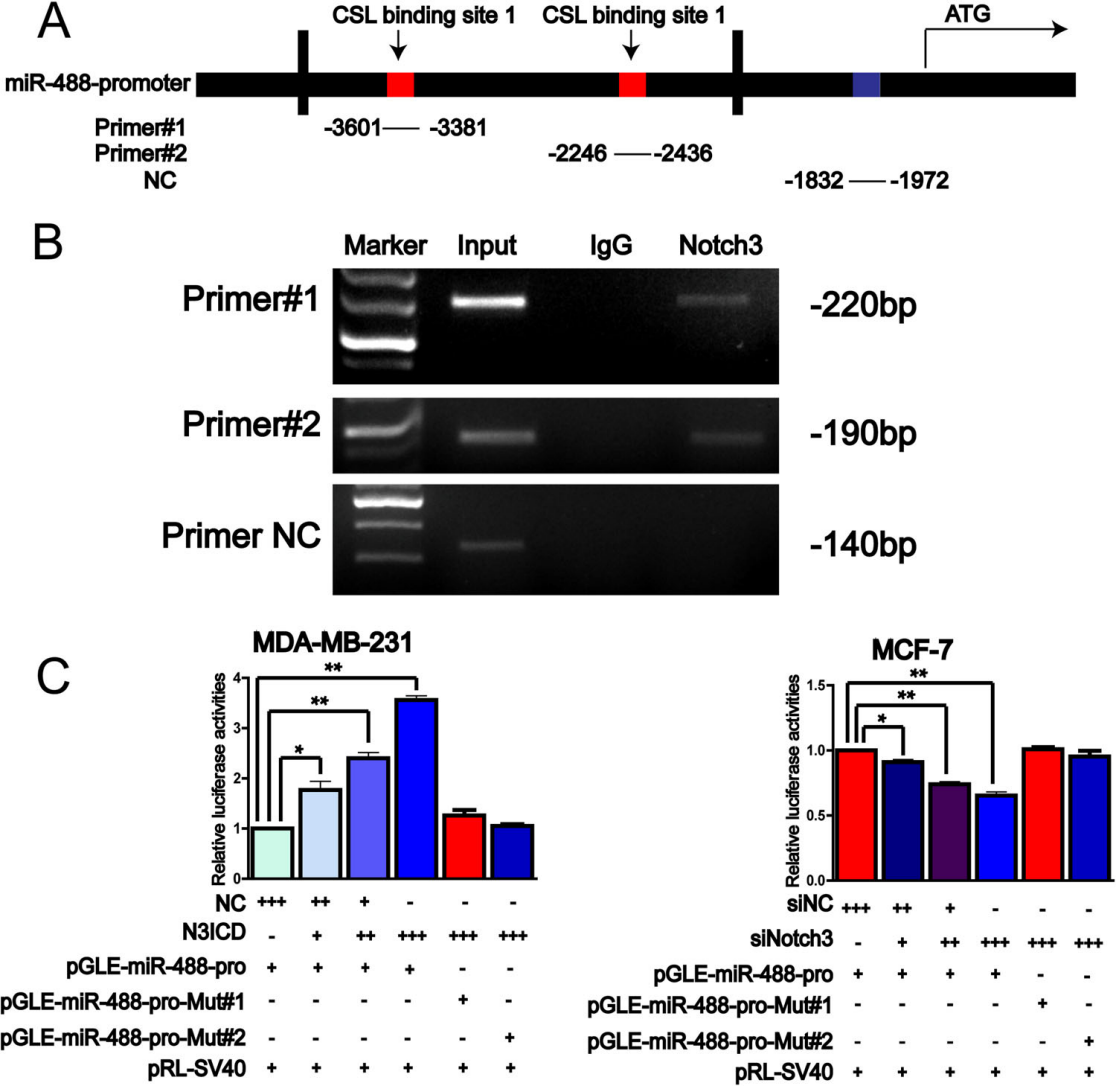
Notch3 directly binds to the promoter region of miR-488 to regulate its expression. **A** The schematic model of the promoter region of miR-488 and its potential Notch/CSL-binding sites. **B** The ChIP results showed that Notch3 directly bind to the Notch/CSL-binding sites and the negative control region did not show any bands in immunoprecipitation. **C** In the reporter gene with the miR-488 promoter, luciferase assays found that the luciferase activities were increased significantly with overexpression of N3ICD in a dose-dependent manner, and decreased dramatically by siNotch3 in a dose-dependent manner. *p < 0.05, **p < 0.01, and ***p < 0.001.

To explore the regulatory factor for the expression of miR-488, ~4000 bp upstream sequence of the miR-488 promoter region were downloaded from the UCSC website and two CSL (CBF1/RBP-Jk/Suppressor of Hairless/LAG-1)-binding sites were found in the promoter of miR-488 (Fig. 6A). Using specific primers for CSL-binding sites and NC, the ChIP assay revealed that Notch3 directly bind to the promoter region of miR-488, containing CSL-binding sites (Fig. 6B).

Further function analyses were conducted through luciferase assay using pGLE-miR-488-pro and corresponding mutant reporter, pGLE-miR-488-pro-Mut. Luciferase assays demonstrated that the luciferase activities produced by pGLE-miR-488-pro were increased with overexpression of the intracellular domain of Notch3 (N3ICD) and decreased with siNotch3 both in a dose-dependent manner, whereas the luciferase activities produced by mutant plasmid were not affected by the overexpression of N3ICD (Fig. 6C).

### The inhibiting function of Notch3 can be reversed by miR-488 inhibitor and conducted through miR-488/FSCN1 axis in breast cancer cells

**Fig. 7:**
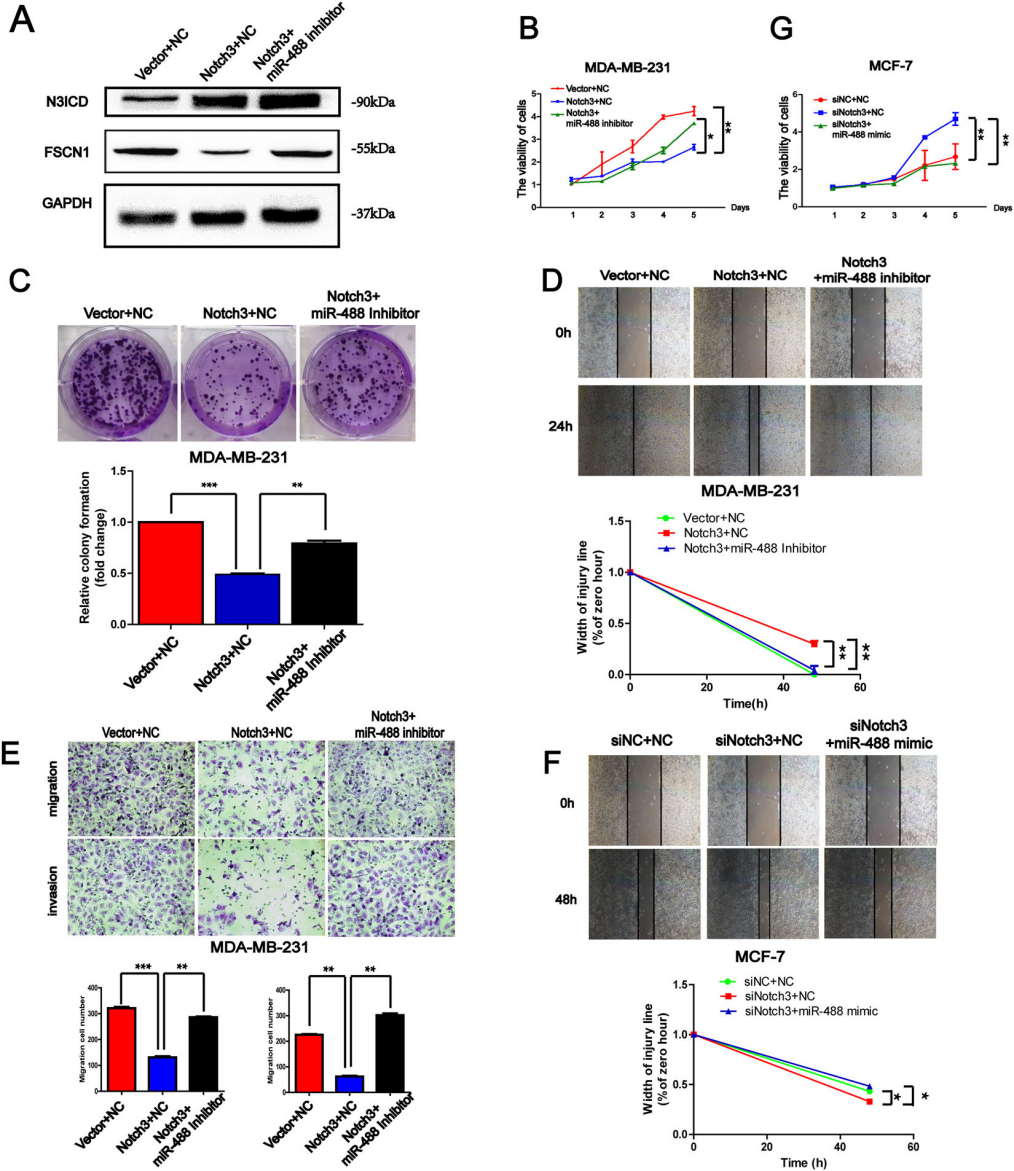
The suppressive function of Notch3 was reversed by suppression of miR-488 level. **A** In the MDA-MB-231 cell, the protein level of FSCN1 was consistent with its mRNA level in the Notch3/miR-488/FSCN1 axis. **B**, **C** The proliferation rate (**B**) and the colony formation (**C**) of MDA-MB-231 were suppressed by Notch3 overexpression, and reversed by miR-488 inhibitor. **D**, **E** The wound healing abilities (**D**) and the motilities in transwell assays (**E**) of MDA-MB-231 were inhibited by Notch3 overexpression, and the suppressive effect was reversed by miR-488 inhibitor. **F**, **G** In MCF-7 cell, siNotch3 significantly promoted the wound healing abilities (**F**) and the proliferation (**G**) of tumor cells through miR-488, while re-overexpression of miR-488 recovered the suppressive function with miR-488 mimic. *p < 0.05, **p < 0.01, and ***p < 0.001.

As the transcriptional factor for regulating miR-488 expression, the functional domain of Notch3, N3ICD significantly increased the expression level of miR-488, and subsequently decreased the mRNA level of FSCN1 (Supplementary Fig. 4). Using the western blot examination, the protein level of FSCN1 was also consistent with its mRNA level and suppressed by increased N3ICD protein (Fig. 7A).

In Fig. 7B, the upregulated Notch3 inhibited the proliferation abilities of MDA-MB-231 cells, and that effect was recovered by a miR-488 inhibitor to promote the proliferation rate of breast cancer cells. And the suppressed colony formation by overexpression N3ICD was also restored by a miR-488 inhibitor (Fig. 7C).

Besides the proliferation abilities of breast cancer cells, the migratory and invasive abilities of MDA-MB-231 were both suppressed by overexpression of N3ICD through wound healing and transwell assays. The reversed efforts were found in breast cancer cells treated by N3ICD overexpression and miR-488 inhibitor at the same time (Fig. 7D, E).

Conversely, siNotch3 dramatically promoted the proliferation, and migration of MCF-7 cells in CCK8 assay and wound healing assay. Artificially increased miR-488 in such cells rescued the inhibition ability, and significantly suppressed the proliferation and migration abilities of MCF-7 cells (Fig. 7F, G).

### The expression of Notch3 was negatively related to FSCN1 levels both in breast cancer cells and in patients with breast cancers

**Fig. 8:**
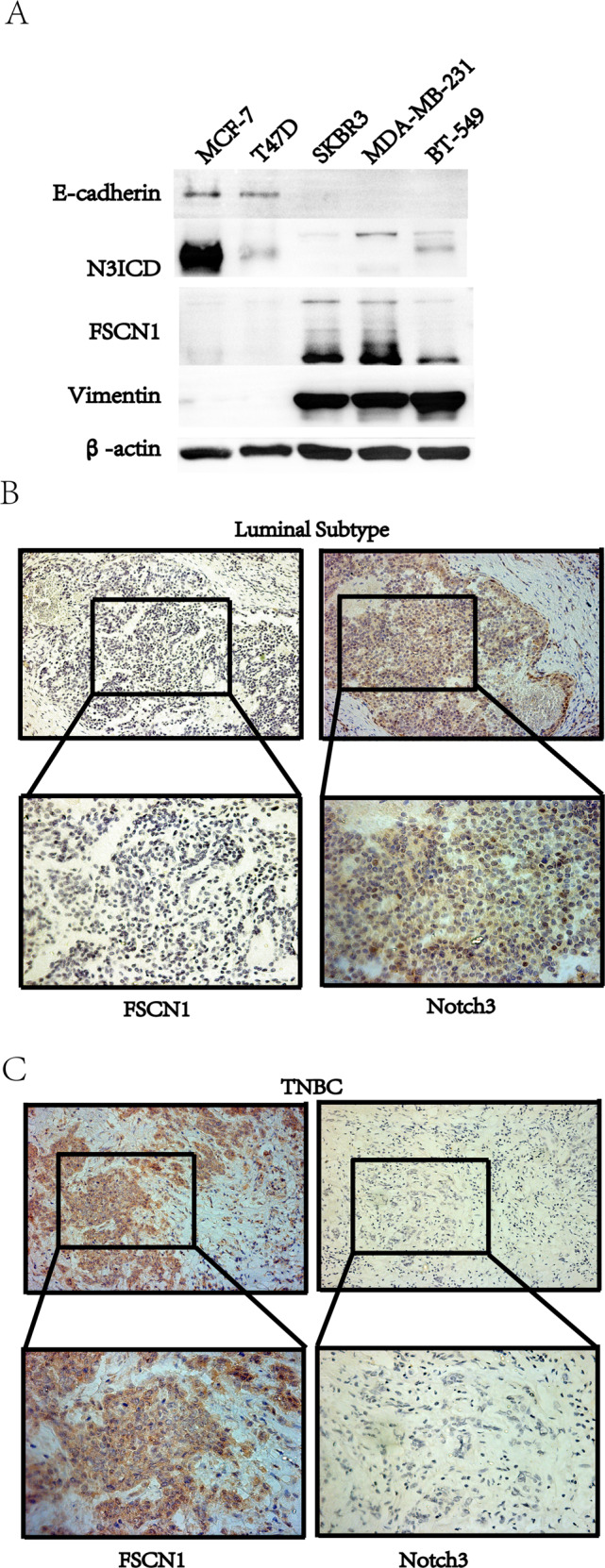
The expression of Notch3 was negatively related to FSCN1 levels both in breast cancer cells and in patients with breast cancers. **A** In different breast cancer cell lines, Notch3 was highly expressed in luminal breast cancer cells and associated with the expression of the epithelial marker, E-cadherin, while the expression of FSCN1 was high in TNBC cells and related to the expression of mesenchymal markers, Vimentin, and Slug. B. The representative images of FSCN1 and Notch3 in patients with a luminal subtype of breast cancers, indicates that the expression of FSCN1 was low, negatively related to high levels of Notch3 in such patients. **C** The representative images of FSCN1 and Notch3 in patients with TNBC, reveals that the expression of FSNC1 is significantly high, also negatively related to low levels of Notch3 in such patients. (Magnification: 200× for the upper one, and 400× for the low one).

In the Notch3/miR-488/FSCN1 axis, the expression of FSCN1 was supposed to be regulated by Notch3 through miR-488 in a negative manner. In Fig. 8A, the expression of Notch3 was high in luminal breast cancer cells and low in TNBC cells related to EMT markers, which was negative to the expression pattern of FSCN1.

To confirm this relationship in the clinic, the samples from patients with breast cancer were collected and examined their expression. According to the immunohistochemistry results, Notch3 localized in both the nuclei and cytoplasm (Fig. 8B), while FSCN1 was exclusively localized in the cytoplasm (Fig. 8C). Notch3 expression was strongly negative associated with FSCN1 levels in breast cancer tissues (Table 1, *r* = −0.248, *p* = 0.010). Both Notch3 and FSCN1 expression were statistically related to the subtypes of breast cancer divided by ER, PR, and HER2 status (Table 2, *p* = 0.001 and 0.012, respectively).

**Table. 1:** The relationship between Notch3 and FSCN1 expression in patients with breast cancer.

Notch3	FSCN1				Spearman	*p* Value
	−	+	++	+++		
−	2	1	0	10	−0.248	0.010
+	8	3	1	0		
++	23	3	2	1		
+++	36	5	7	4

**Table. 2:** The correlation of Notch3 and FSCN1 expression with the subtypes of breast cancers.

Subtypes	Notch3^−^ (*n* = 13)	Notch3^+^ (*n* = 93)	*p* Value	FSCN1− (*n* = 77)	FSCN1^+^ (*n* = 29)	*p* Value
Luminal	3 (4.7%)	61 (95.3%)	0.001	46 (71.8%)	18 (28.2%)	0.012
HER-2	5 (16.7%)	25 (83.3%)		26 (86.7%)	4 (13.3%)	
TNBC	5 (41.7%)	7 (58.3%)		5 (41.7%)	7 (58.3%)	

## Discussion

The primary findings of this current study provide critical insights into the crucial role of the Notch3/miR-488/FSCN1 axis in EMT and metastasis of breast cancer. It was found that the increased Notch3 contributed to increased expression of miR-488, which further suppressed the expression of FSCN1, and corresponding proliferation, migration, and invasion of breast cancer cells. Thus, the current results uncover a novel molecular mechanism of the Notch3/miR-488/FSCN1 axis in breast carcinomas.

As a novel miRNA, the research focused on miR-488 was limited and reported the downregulation of miR-488 in several human cancers and its low expression significantly correlated with progression, metastasis, and poor survival^5–9^, indicating the tumor-suppressive role of miR-488 in different types of cancers. However, the function and mechanism of miR-488 in breast cancer is still unclear. This study first reported the suppressive role of miR-488 in the proliferation, migration, and invasion abilities of breast cancer cells. Upregulating miR-488 inhibited while silencing miR-488 enhanced EMT process, confirmed by the expression of epithelial/mesenchymal markers.

To further explore the underlying mechanism for the downregulation of miR-488 in breast cancer remains unclarified. Based on the online prediction tools, the potential downstream genes were collected and analyzed with their reported functions. It is accepted that the actin and microtubule cytoskeletons form highly versatile, dynamic polymers to organize cytoplasmic organelles and intracellular compartments, define cell polarity and generate forces during the cell cycles^19^. During the cell migration, they generate protrusive forces at the front and retraction forces at the rear, so the abnormal cytoskeletal structure affects all aspects of cell behavior, especially the cell motility and metastasis in cancer^20^. Actin aggregation under the plasma membrane provides the main force for cell movement, and the factors promoting mitochondrial aggregation and cytoskeletal disruption can promote migration and invasion properties of a breast cancer cell line^21,22^. Actin-binding protein, FSCN1, predicted as the potential downstream of miR-488 in our research is reported to regulate cell adhesion, coordinate cell movement, and cytoskeleton^23^.

Emerging shreds of evidence indicate FSCN1 possessing a causal role in the development and metastasis of several types of cancers^24^. In clinical tissues, strong positive FSCN1 expression was demonstrated as a novel diagnostic marker of aggressive TNBC^25^. Interestingly, Wang et al. revealed that the epidermal growth factor (EGF) induced the expression of FSCN1 through activation of MAPK (mitogen-activated protein kinase), which subsequently promoted the migration and invasion of TNBC cells, and the expression of FSCN1 was significantly decreased with the co-treatment of siFSCN1 and Gefitinib (EGFR inhibitor), compared with the single treatment of siFSCN1 or Gefitinib, followed with significant suppression of migration and invasion abilities of TNBC cells^26^. They suggested co-targeting EGFR and FSCN1 dual biomarker as a novel therapeutic strategy for TNBC. And the current results provided another method to suppress the expression of FSCN1 through the tumor-suppressive function of miR-488 by directly binding to the 3′UTR region of FSCN1.

To complete the mechanism of miR-488 in breast cancer, the promoter region of miR-488 was downloaded and analyzed to predict the transcriptional factor involved in regulating the expression of miR-488, and the Notch/CSL-binding sites were found in this region. Previous studies showed that Notch3 plays a tumor-suppressive role in the development of breast cancer, which is positively associated with ERα expression in breast cancer, and upregulating Kibra level to inhibit Hippo/YAP (Yes-associated protein) pathway, resulting in the inhibition of proliferation and invasion of breast cancer^17,27^. On the other hand, an inhibitor of differentiation 2 activated Notch3 expression by blocking E2A binding to the E-box motif of the Notch3 promoter region, followed by inhibition of EMT process in breast cancer^28^. The current study further explored the suppressive mechanism of Notch3 and found that miR-488 is the downstream factor of Notch3, regulating in transcriptional level by directly binding to the Notch/CSL-binding site of the miR-488 promoter. Importantly, the regulating effect of miR-488 can be modulated by the expression of Notch3, to regulate the proliferation and motility of breast cancer cells.

The miRNAs are a set of noncoding RNAs, involved in various aspects of the occurrence and development of cancers. The current research extends the role of miR-488 in the development of breast cancer and uncovered the underlying mechanism involved in the function of miR-488. As a conclusion, miR-488 is provided as a novel biomarker for inhibition of the development of breast cancer, through inhibiting the abnormal cytoskeletal structure with the downregulation of FSCN1 expression, which can be promoted by the transcriptional factor, Notch3, providing the Notch3/miR-488/FSCN1 axis as novel treatment strategies for the patients with breast cancer.

## Supplementary information

Supplementary Figure Legends

Supplementary Figure 1

Supplementary Figure 2

Supplementary Figure 3

Supplementary Figure 4

## References

[1] Anastasiadi Z, Lianos GD, Ignatiadou E, Harissis HV, Mitsis M (2017). Breast cancer in young women: an overview. Updates Surg..

[2] Peart OMetastatic (2017). Breast cancer. Radio. Technol..

[3] Bertozzi N, Pesce M, Santi PL, Raposio E (2017). Oncoplastic breast surgery: comprehensive review. Eur. Rev. Med. Pharm. Sci..

[4] Sikand K, Slaibi JE, Singh R, Slane SD, Shukla G (2011). C. miR 488* inhibits androgen receptor expression in prostate carcinoma cells. Int J. Cancer.

[5] Fang C (2017). MiR-488 inhibits proliferation and cisplatin sensibility in non-small-cell lung cancer (NSCLC) cells by activating the eIF3a-mediated NER signaling pathway. Sci. Rep..

[6] Hu D (2017). MiR-488 suppresses cell proliferation and invasion by targeting ADAM9 and lncRNA HULC in hepatocellular carcinoma. Am. J. Cancer Res..

[7] Li N (2017). MicroRNA-488-3p sensitizes malignant melanoma cells to cisplatin by targeting PRKDC. Cell Biol. Int..

[8] Shi B, Yan W, Liu G, Guo Y (2018). MicroRNA-488 inhibits tongue squamous carcinoma cell invasion and EMT by directly targeting ATF3. Cell Mol. Biol. Lett..

[9] Masuda T (2018). Circulating pre-microRNA-488 in peripheral blood is a potential biomarker for predicting recurrence in breast cancer. Anticancer Res..

[10] Dang L, Yoon K, Wang M, Gaiano N (2006). Notch3 signaling promotes radial glial/progenitor character in the mammalian telencephalon. Dev. Neurosci..

[11] Zeng FM (2017). Fascin phosphorylation sites combine to regulate esophageal squamous cancer cell behavior. Amino Acids.

[12] Liu J (2018). Collagen 1A1 (COL1A1) promotes metastasis of breast cancer and is a potential therapeutic target. Discov. Med..

[13] Liu W, Wang X (2019). Prediction of functional microRNA targets by integrative modeling of microRNA binding and target expression data. Genome Biol..

[14] Agarwal, V., Bell, G. W., Nam, J. W. & Bartel, D. P. Predicting effective microRNA target sites in mammalian mRNAs. *Elife* **4**, e05005 (2015). 10.7554/eLife.05005 PMC4532895 26267216

[15] Raney BJ (2014). Track data hubs enable visualization of user-defined genome-wide annotations on the UCSC Genome Browser. Bioinformatics.

[16] Hu H (2019). AnimalTFDB 3.0: a comprehensive resource for annotation and prediction of animal transcription factors. Nucleic Acids Res..

[17] Dou XW (2017). Notch3 maintains luminal phenotype and suppresses tumorigenesis and metastasis of breast cancer via trans-activating estrogen receptor-alpha. Theranostics.

[18] Liu J, Shen JX, Hu JL, Huang WH, Zhang GJ (2014). Significance of interleukin-33 and its related cytokines in patients with breast cancers. Front. Immunol..

[19] Hall A (2009). The cytoskeleton and cancer. Cancer Metastasis Rev..

[20] Zheng Q (2015). X-ray radiation promotes the metastatic potential of tongue squamous cell carcinoma cells via modulation of biomechanical and cytoskeletal properties. Hum. Exp. Toxicol..

[21] Lee MW (2014). The CT20 peptide causes detachment and death of metastatic breast cancer cells by promoting mitochondrial aggregation and cytoskeletal disruption. Cell Death Dis..

[22] Sanchez AM (2016). LH and FSH promote migration and invasion properties of a breast cancer cell line through regulatory actions on the actin cytoskeleton. Mol. Cell Endocrinol..

[23] Li X, Law JW, Lee AY (2012). Semaphorin 5A and plexin-B3 regulate human glioma cell motility and morphology through Rac1 and the actin cytoskeleton. Oncogene.

[24] Tan VY, Lewis SJ, Adams JC, Martin RM (2013). Association of fascin-1 with mortality, disease progression and metastasis in carcinomas: a systematic review and meta-analysis. BMC Med..

[25] Wang CQ (2016). Fascin-1 as a novel diagnostic marker of triple-negative breast cancer. Cancer Med..

[26] Wang CQ (2017). EGFR conjunct FSCN1 as a novel therapeutic strategy in triple-negative breast cancer. Sci. Rep..

[27] Zhang X (2016). Notch3 inhibits epithelial-mesenchymal transition by activating Kibra-mediated Hippo/YAP signaling in breast cancer epithelial cells. Oncogenesis.

[28] Wen XF (2018). Inhibitor of DNA binding 2 inhibits epithelial-mesenchymal transition via up-regulation of Notch3 in breast cancer. Transl. Oncol..

