# Supplementary material for: MicroRNA-488 inhibits proliferation and motility of tumor cells via downregulating FSCN1, modulated by Notch3 in breast carcinomas
Source: Cell Death Dis. 2020 Oct 24;11(10):912. doi: 10.1038/s41419-020-03121-5 (PMC7585581; doi:10.1038/s41419-020-03121-5)
Supplement: Supplementary file 1 — Supplementary Figure Legends [file 41419_2020_3121_MOESM1_ESM.docx]

**Supplementary Tables**

**Table S1. The sequences of oligonucleotides**

| **Name** | **Sequence (5’-3’)** |
| --- | --- |
| miR-488 mimic | UUGAAAGGCUAUUUCUUGGUC |
| miR-488 inhibitor | UUGAAAGGCUAUUUCUUGGUC |
| NC | UUCUCCGAACGUGUCACGUTT |
| siNotch3-1 | CACCUAUAACUGCCAGUGC |
| siNotch3-2 | UAUAGGUGUUGACGCCAUCCACGCA |
| siFSCN1-1 | GAGCAUGGCUUCAUCGGCU |
| siFSCN1-2 | CACGGGCACCCUGGACGCCAA |
| siNC | UUCUCCGAACGUGUCACGUTT |

**Table S2. The sequences of primers**

| **Gene** | **Primer** | **Sequence (5’-3’)** |
| --- | --- | --- |
| Notch3 | F | ATGCAGGATAGCAAGGAGGA |
|  | R | AAGTGGTCCAACAGCAGCTT |
| FSCN1 | F | CCAGGGTATGGACCTGTCTG |
|  | R | GTGTGGGTACGGAAGGCAC |
| β-actin | F | GCTGATGCTGAAGTGTGGTG |
|  | R | AGCGAGCATCCCCCAAAGTT |
| ChIP site 1 | F | CTGGCGCAGGGTTCTTCT |
|  | R | CCTCCCTTGCCACAGAGAAT |
| ChIP site 2 | F | AAGAGGCCAGCAGCTCTCAG |
|  | R | GGCCCACCCTCACAAAGA |
| ChIP NC | F | AGCGTCAATCCTTTGAGGGC |
| Hsa-miR-488-3p | R  F  R | GAGGACGTCGGAGATTTGCC  GTTGAAAGGCTATTTCTTGGT  AGTGCAGGGTCCGAGGTATT |

**Table S3. The information of antibodies**

| **Antibody** | **Company/Catalogue number** | **Species** | **Application (Dilution)** |
| --- | --- | --- | --- |
| Notch3 | CST/5276S | Rabbit/mono | WB (1:3000)/IP (1:500)/IHC (1:200) |
| FSCN1 | CST/55K-2 | Mouse/mono | WB(1:3000)/IHC (1:200) |
| E-cadherin | DAKO/NCH-38 | Mouse/mono | WB (1:3000) |
| Vimentin | CST/5741S | Rabbit/mono | WB (1:1000) |
| Snail | CST/C15D3 | Rabbit/mono | WB (1:3000) |
| Slug | CST/C19G7 | Rabbit/mono | WB (1:2000) |
| GAPDH | Santa Cruz/sc-32233 | mouse/mono | WB (1:3000) |
| β-actin | Zsbio/PR-0255 | Mouse/mono | WB (1:3000) |
| IgG | Santa Cruz/sc-66931 | Rabbit/poly | IP (1:500) |

**Supplementary Figures**

**Figure S1. The colony formation was regulated by miR-488 in breast cancer cells.** A. The colony formation of BT-549 was suppressed by miR-488 mimic, similar with that in MDA-MB-231. B. MiR-488 inhibitor increased the colony formation of T47D cells.

**Figure S2. FSCN1 inhibits the motility of breast cancer cells via the EMT process.** A. The siRNA technology was used to knockdown the expression of FSCN1. B. Suppressing FSCN1 expression significantly reverse the EMT process with increased E-cadherin expression and decrease Vimentin and Slug levels in MDA-MB-231 cells. C. The proliferation rate of MDA-MB-231 cells was suppressed by decreased FSCN1 levels in CCK8 assays. D. The colony formation of MDA-MB-231 and BT-549 cells were both inhibit by low FSCN1 levels. E. The migration and invasion abilities of MDA-MB-231 cells were decreased in siFSCN1-treated MDA-MB-231 cells compared with the NC group. * means *p* < 0.05, ** means *p* < 0.01, *** means *p* < 0.001.

**Figure S3. The expression of FSCN1 was recovered by miR-488 inhibitor, suppressed by siFSCN1, and confirmed by Western Blot in MCF-7 cells.**

**Figure S4. The suppressive function of Notch3 on FSCN1 was reversed by suppression of miR-488 level.** In MDA-MB-231 cells, the mRNA of FSCN1 expression was suppressed by Notch3 through miR-488 and miR-488 inhibitor reversed the suppressive effect of Notch3 to upregulate FSCN1 expression. * means *p* < 0.05, ** means *p* < 0.01, *** means *p* < 0.001.
