# Supplementary figures and images for: MicroRNA-488 inhibits proliferation and motility of tumor cells via downregulating FSCN1, modulated by Notch3 in breast carcinomas
Source: Cell Death Dis. 2020 Oct 24;11(10):912. doi: 10.1038/s41419-020-03121-5 (PMC7585581; doi:10.1038/s41419-020-03121-5)

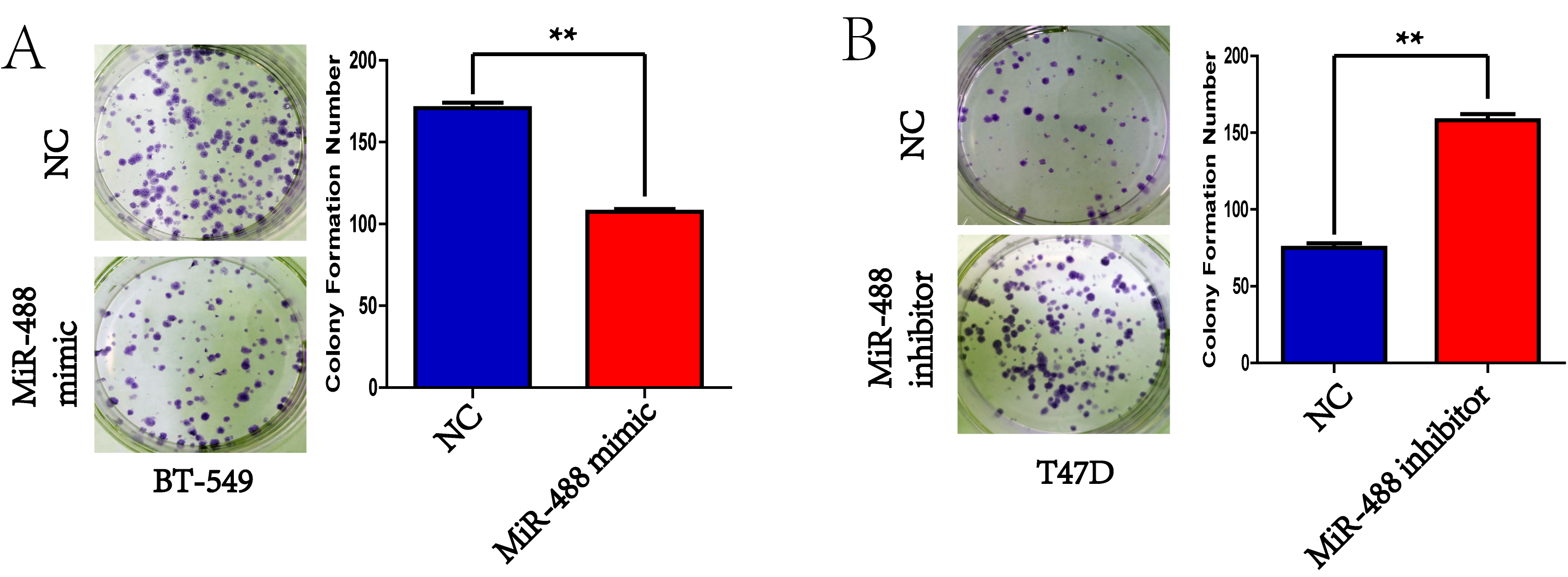

Supplement: Supplementary file 2 — Supplementary Figure 1 [file 41419_2020_3121_MOESM2_ESM.tif]

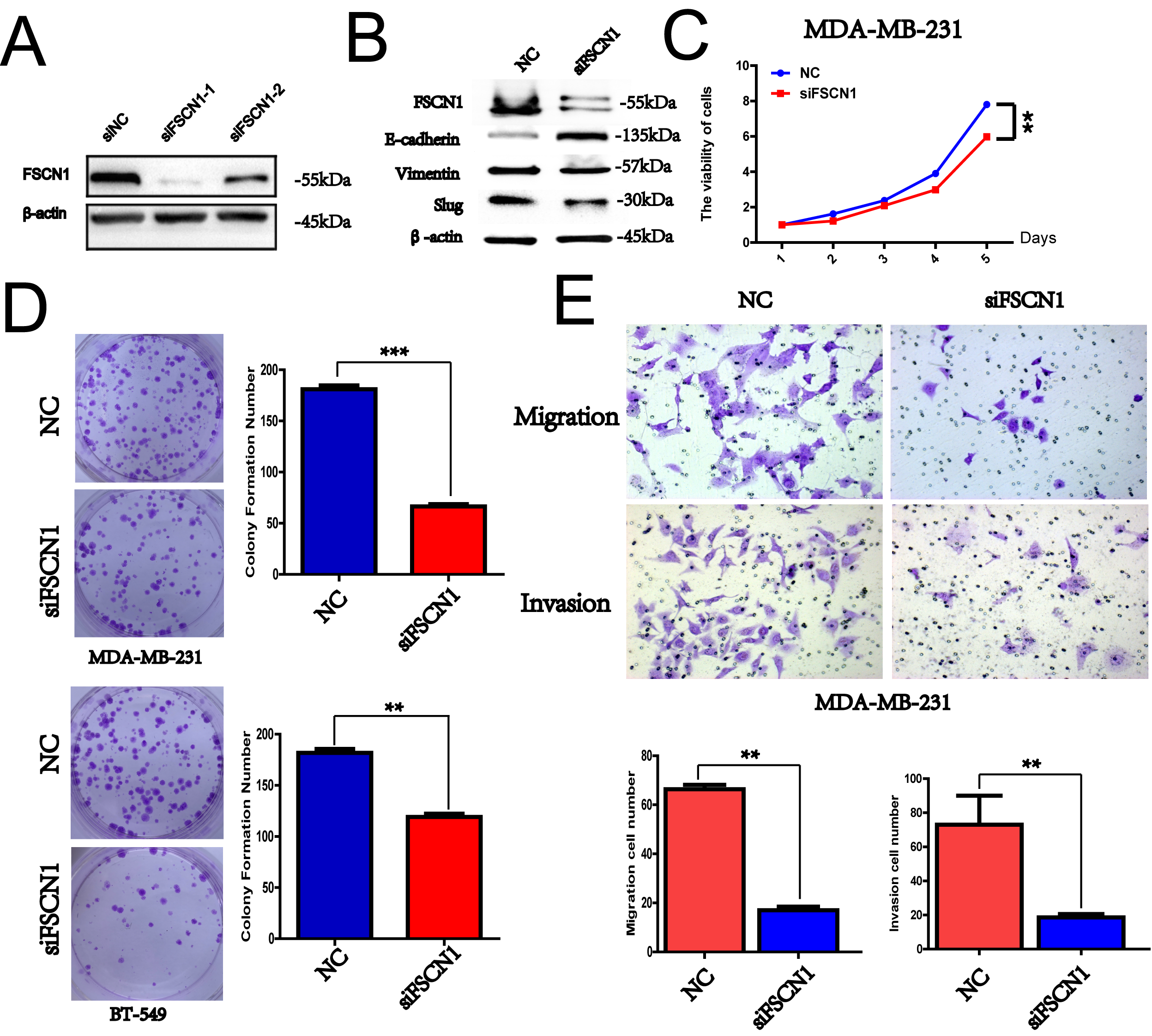

Supplement: Supplementary file 3 — Supplementary Figure 2 [file 41419_2020_3121_MOESM3_ESM.tif]

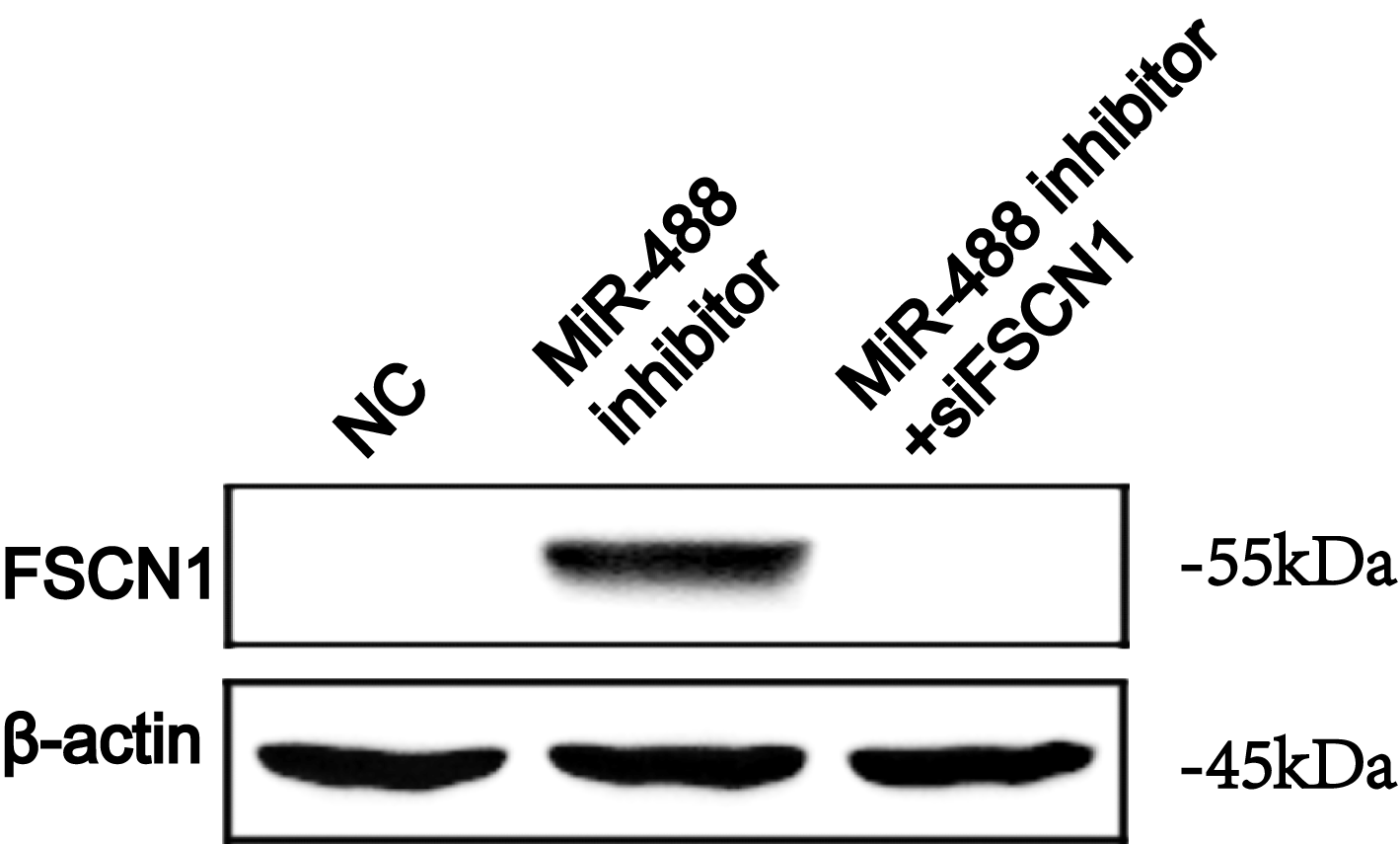

Supplement: Supplementary file 4 — Supplementary Figure 3 [file 41419_2020_3121_MOESM4_ESM.tif]

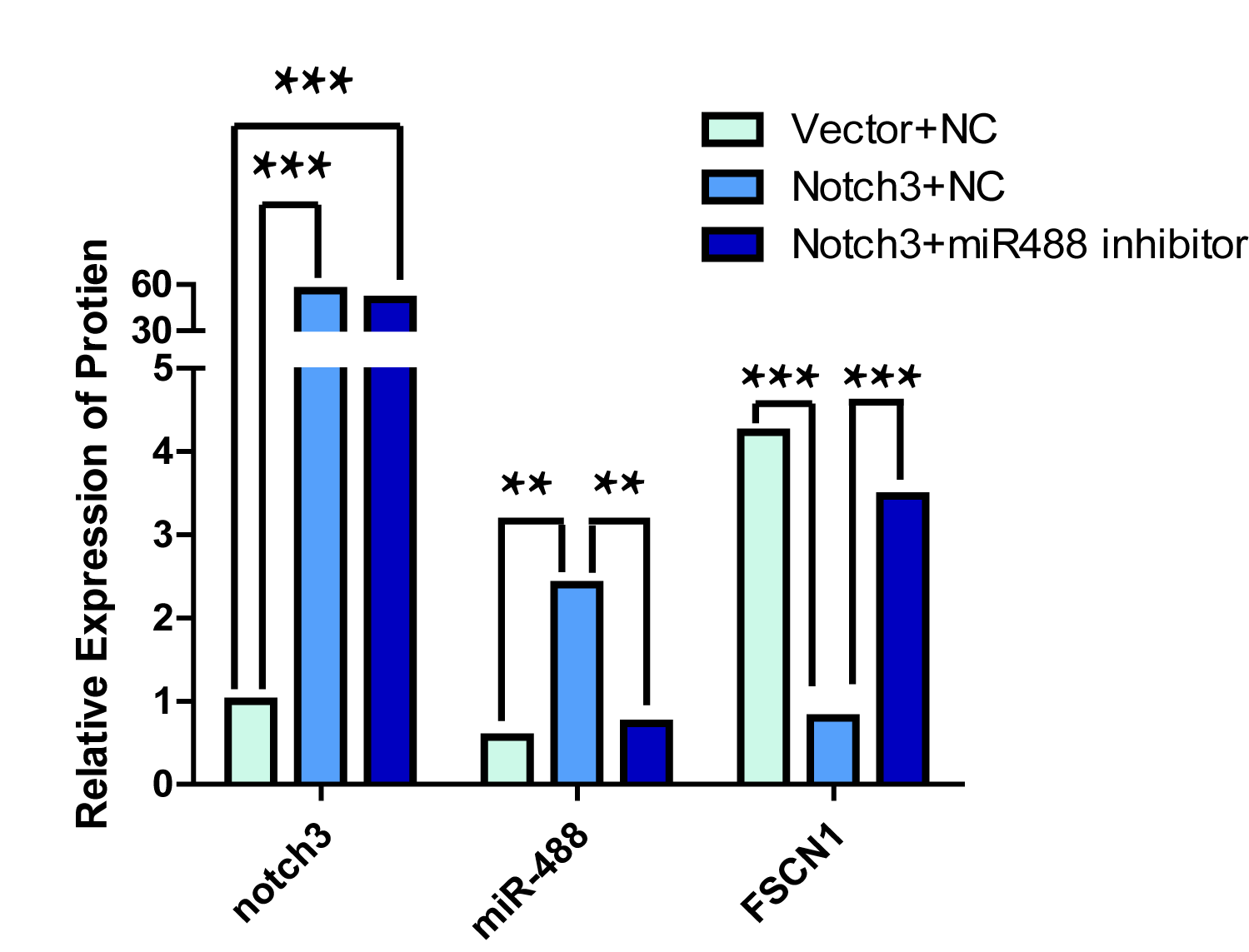

Supplement: Supplementary file 5 — Supplementary Figure 4 [file 41419_2020_3121_MOESM5_ESM.tif]
